# Supplementary material for: Resolving the full spectrum of human genome variation using Linked-Reads
Source: Genome Res. 2019 Apr;29(4):635–45. doi: 10.1101/gr.234443.118 (PMC6442396; doi:10.1101/gr.234443.118)
Supplement: Supplemental Material [file supp_gr.234443.118_Supplemental_longranger-2.2.zip › longranger-2.2.tar.bz2!/lr2.2-public/lib/go/src/code.google.com/vitess/doc/slides/Vitess2014.pdf]

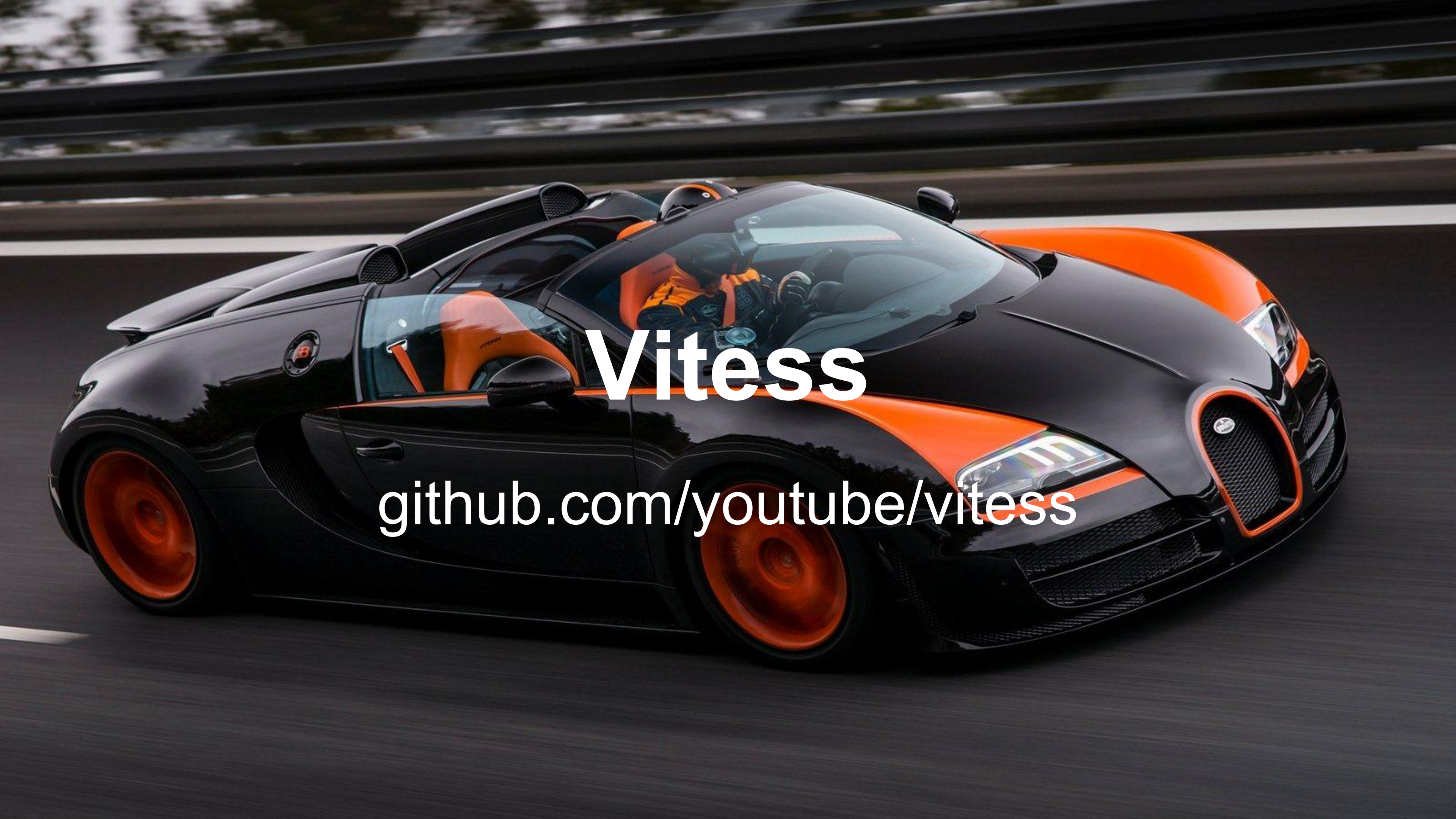

Vitess

github.com/youtube/vitess

What is Vitess

- A scalable and efficient storage solution for the web
- Tools
  - Clone, remaster, sharding, split, etc.
- Servers
  - Optimize MySQL performance

The sweet spot

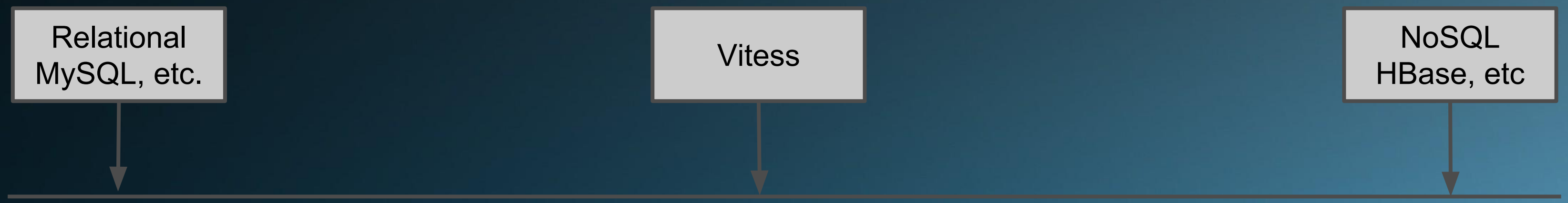

- Pros
  - Transactions
  - Indexes
  - Joins
- Cons
  - No sharding
  - ACID
  - Schema

- Pros
  - Transactions (limited)
  - Indexes
  - Joins
  - Sharding
- Cons
  - Eventual consistency
  - Schema

- Pros
  - Sharding
  - Unstructured data
- Cons
  - Eventual consistency
  - No transactions
  - No indexes
  - No joins

Why tools?

- Start off simple
- Issues:
    - Data size
    - QPS
    - Uptime
    - Backups
    - Crash recovery
    - Number of connections
        - Let’s ignore this for now

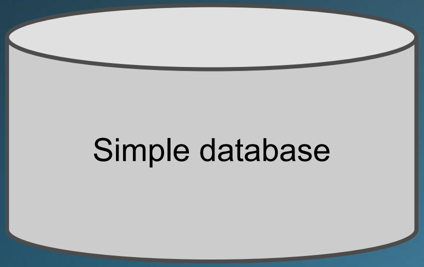

Add replication

- Issues
  - Utilization
- And
  - Data size
  - Write QPS
  - Write uptime
  - ~~Backups~~
  - ~~Read Uptime~~
  - ~~Read QPS~~
  - ~~Crash recovery~~

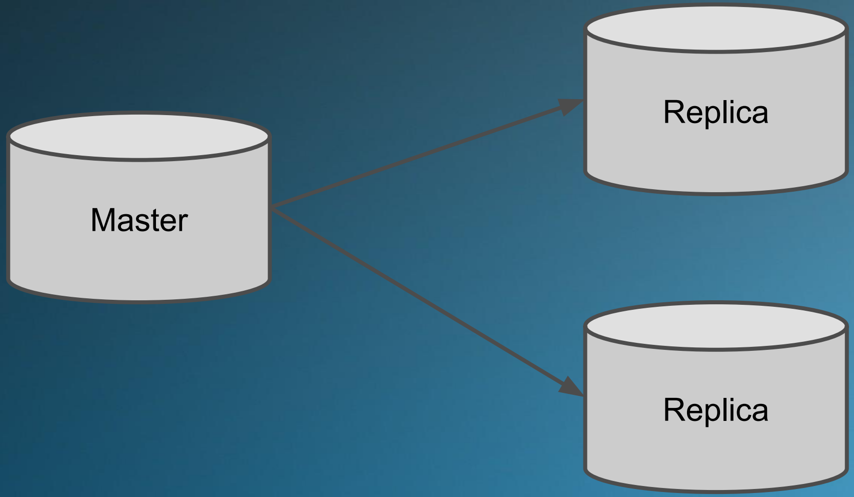

Add categories

- Issues
  - Track categories
- And
  - Data size
  - Write QPS
  - Write uptime
  - ~~Utilization~~

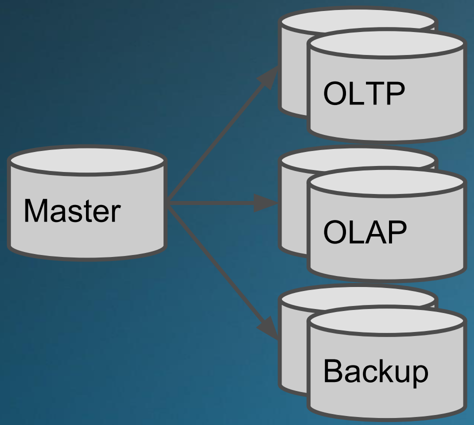

Add sharding

- Issues
  - Complex client
  - Resharding workflow
  - Track shards
  - Track replication
  - Disaster recovery
- And
  - Track categories
  - Write uptime
  - ~~Data size~~
  - ~~Write QPS~~

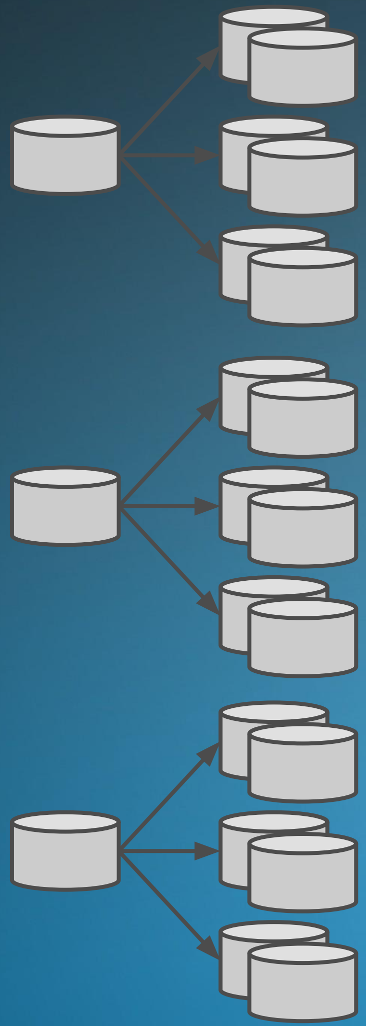

Add data centers

- Issues
  - Failover workflow
  - Number of connections
- And
  - Complex client
  - Resharding workflow
  - Track shards
  - Track replication
  - Track categories
  - Write uptime
  - ~~Disaster recovery~~

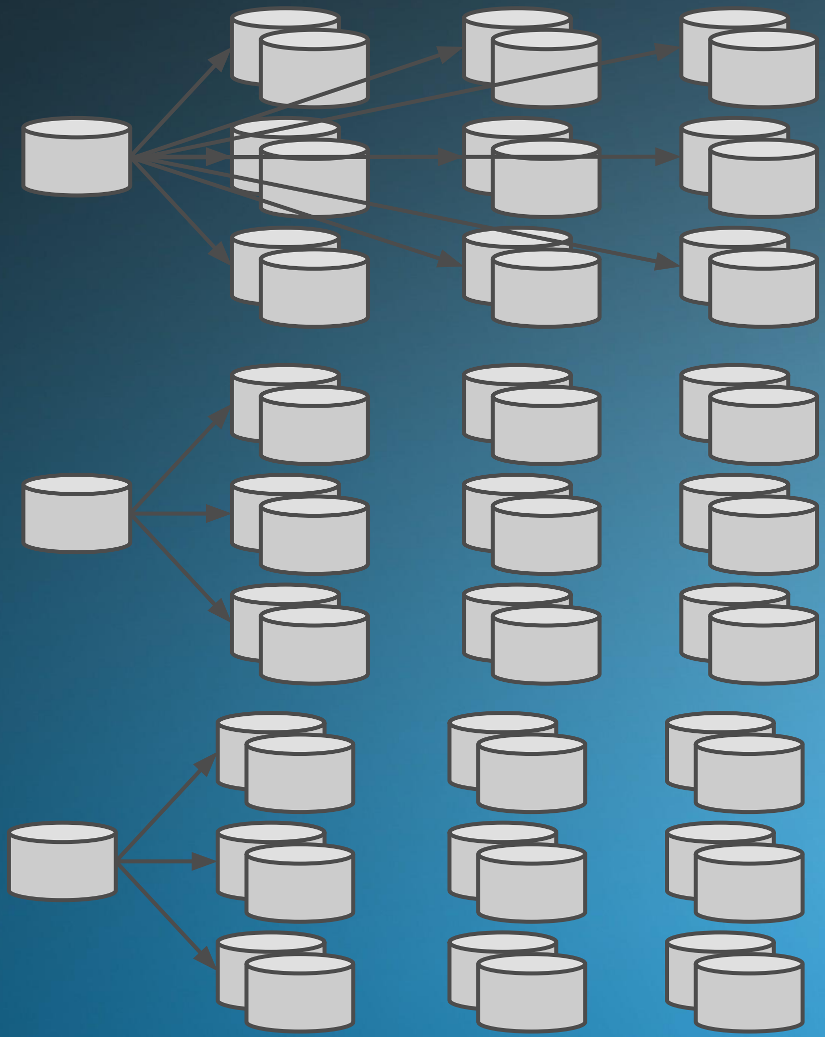

More complexity

- Multiple logical databases (keyspaces)
- Lookup database

Vitess lock server (zookeeper)

- Global info
  - Keyspaces
  - Shard graph
  - Master databases
  - List of data centers
- Per-data center
  - Replicas
  - Categories
  - Serving graph

Vitess tools

- vtctl command-line tool
  - Failover workflow
  - Resharding workflow
  - Track shards
  - Track replication
  - Track categories
- vtctld web server
  - Topology browser

Vitess servers

- vttablet
  - Sits in front of MySQL and mediates all queries
  - Pools connections
  - Performs local management tasks requested by vtctl
- vtgate query server
  - Complex client
  - Failover workflow
  - Resharding workflow

The big picture

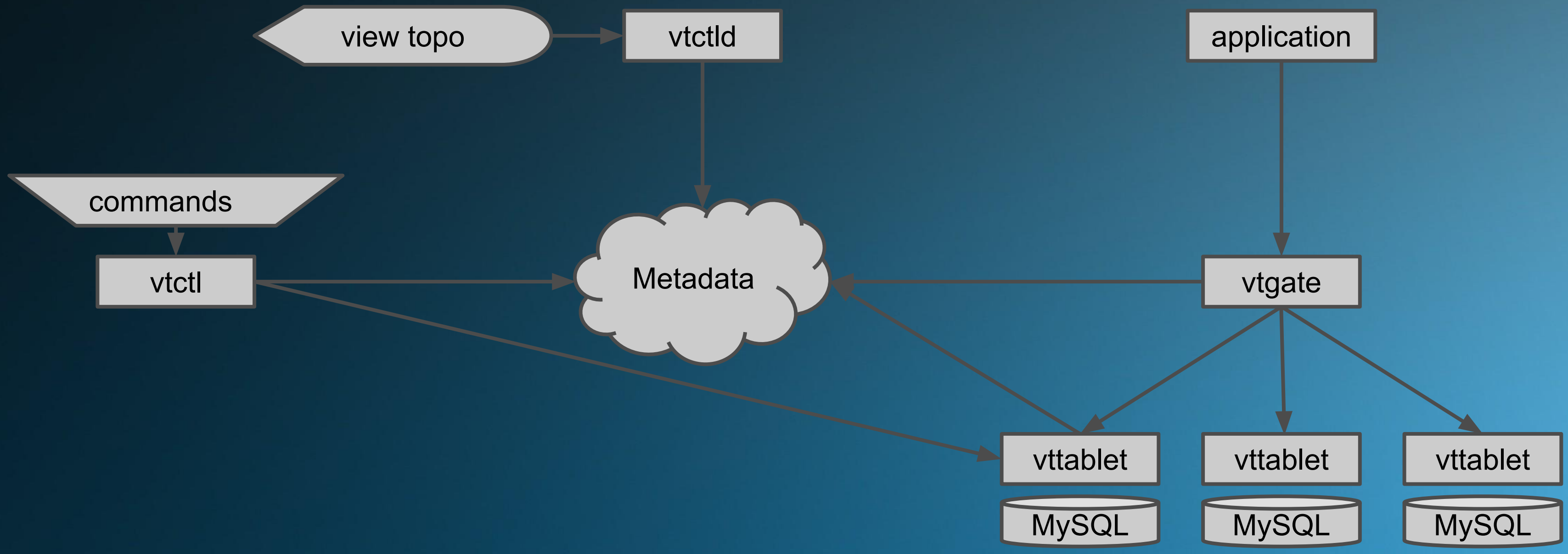

vitess journey

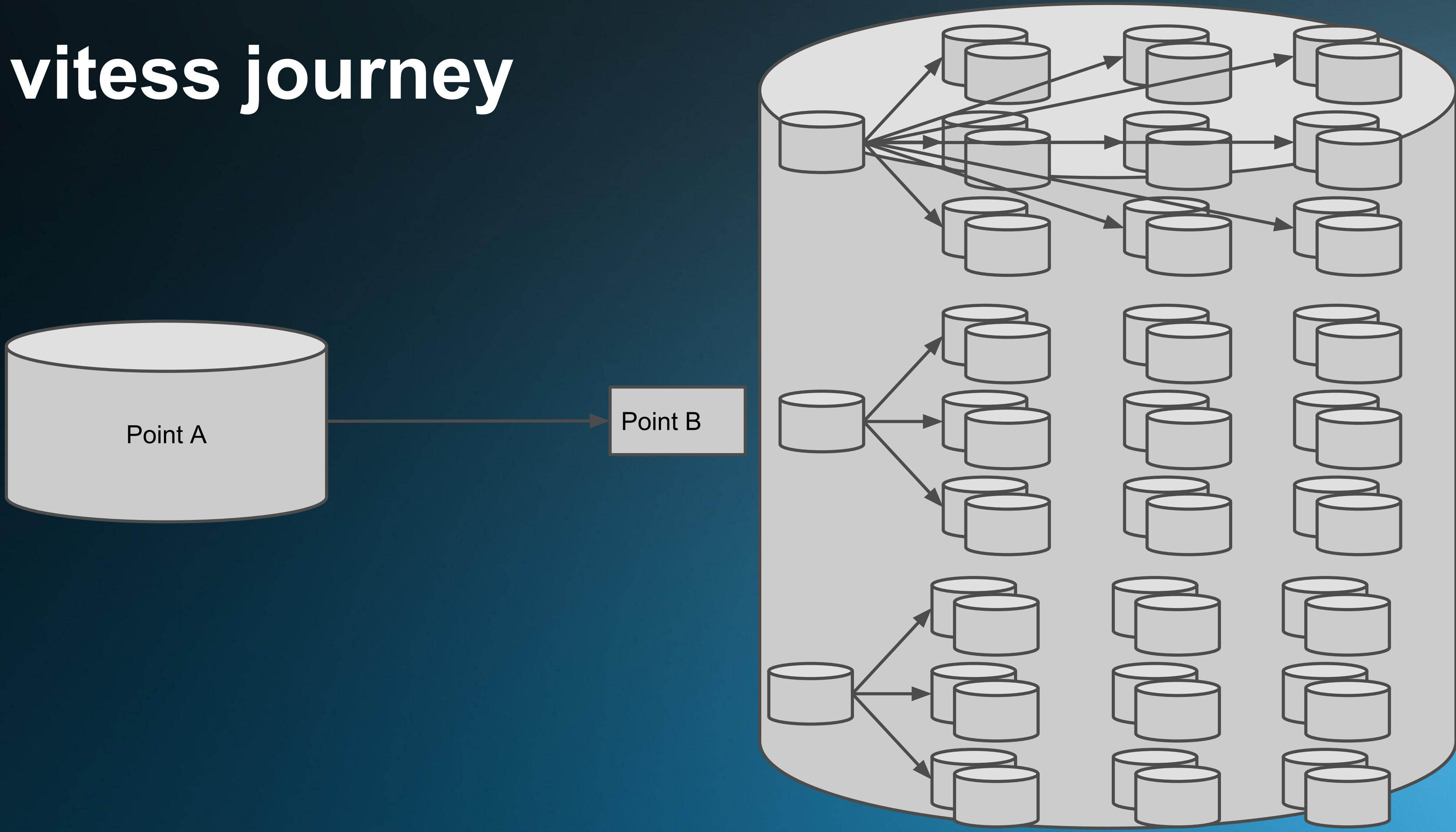

Why servers?

- MySQL shortcomings
  - Cost of connections
  - No throttling
  - Unbounded results
  - Buffer cache
  - No blacklisting
  - Insufficient stats & diagnostics
- Sharding abstraction
  - Query routing
  - Results aggregation
  - Joins
  - Index lookups
  - Retries

vttablet: Turbo-charging MySQL

- Co-exists with every MySQL instance
- Query service
- Tablet management
- Binlog streaming services

Query service: efficiency

- Connection pooling
- Results reuse
- SQL parser, query rewriter
- Rowcache
  - Primary key based fetches
  - Consistent
  - More efficient than MySQL buffer cache
  - Used for subquery lookups

Query service: failsafes

- Transaction management
- Row count limit
- Transaction limit
- Query and transaction timeouts
- Query blacklisting

Query service: enrichment

- DML annotations
- Stats
- Info & error logs
- Verbose streamlog
- Zero downtime restarts

vttablet submodules

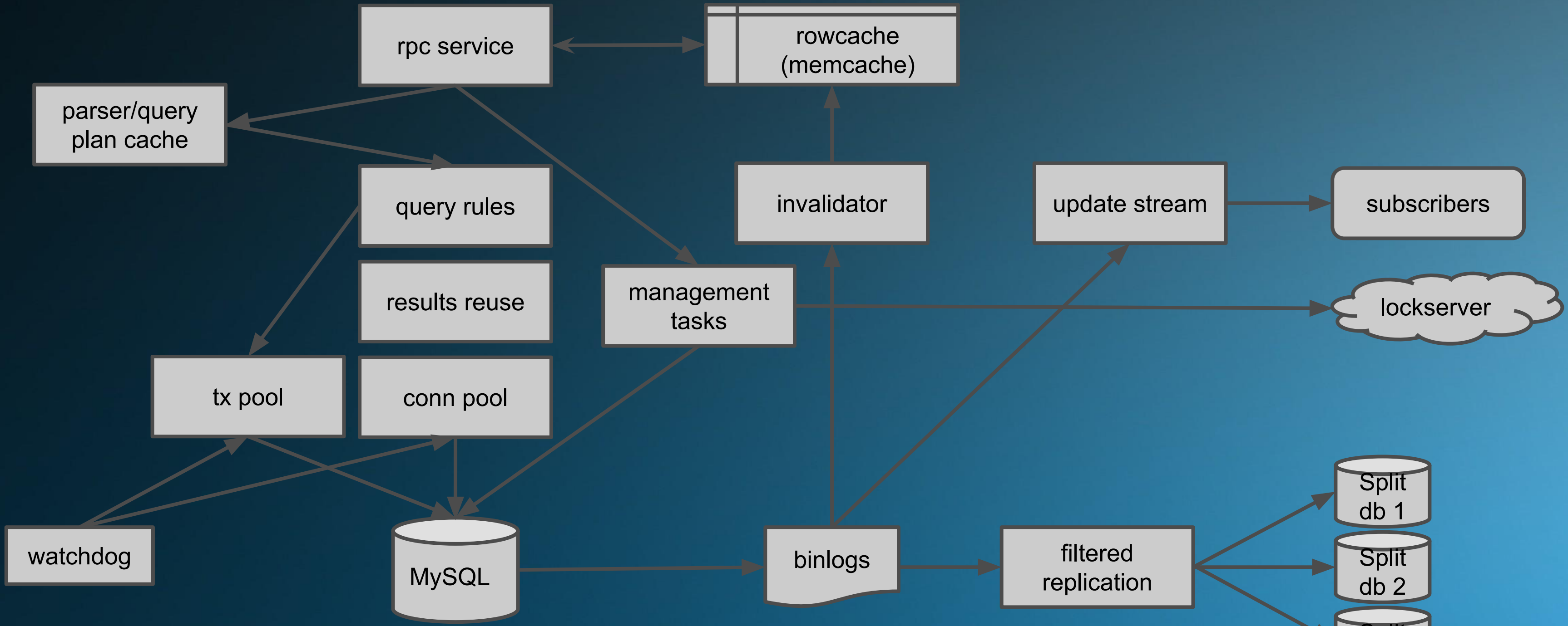

Production ready

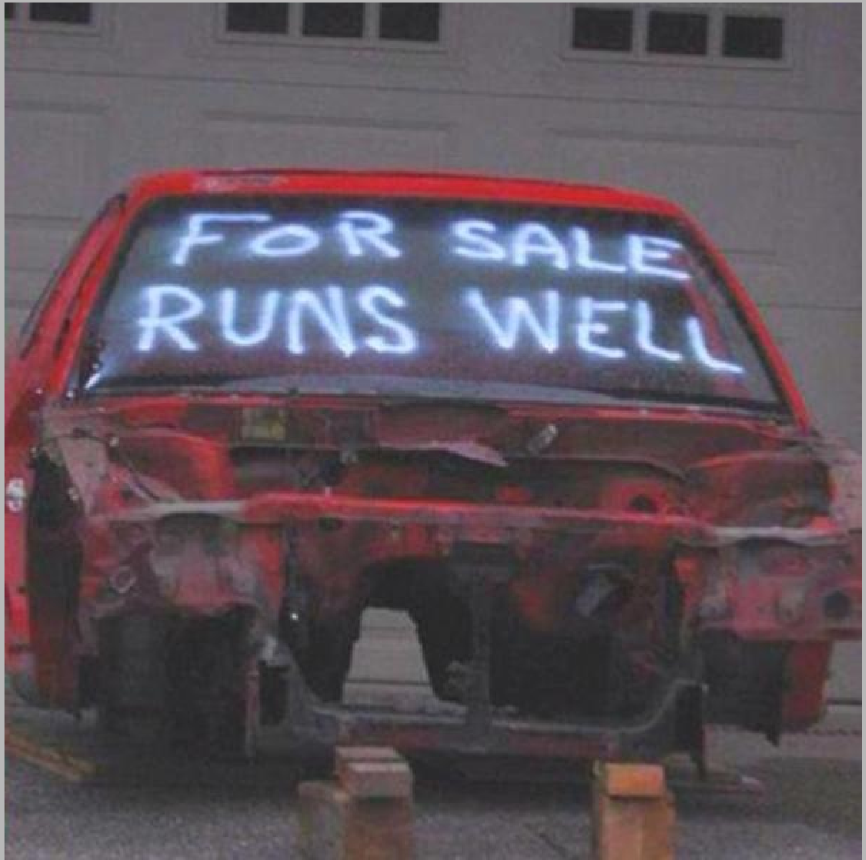

vttablet in production

- Serves all of YouTube's MySQL traffic
- In production since 2011
- 10K-40K connections
- Vast increase in MySQL serving capacity
- Config, logging, stats & monitoring
- Rowcache getting ready to launch

Dependencies

- Google MySQL (group id)
- Zookeeper
- Go 1.2
- Python
- Other misc Linux tools (apt-get)

vtgate

- Route client queries to the right vttablet
- Balance load
- Built-in retries
- Up-to-date with db state changes
- Multi-shard queries
- Multi-db transactions

vtgate future

- Cross-shard indexes
- Cross-shard aggregations
- Cross-db joins
- Map-reduce
- Resumable streaming queries

RPC protocol: rpcplus

- Fork of go's rpc
  - Call context
  - Streaming
- Security
  - SASL Authentication
  - SSL
- Encoding agnostic
  - bson, gob, json
  - Other encodings
- Clients
  - go
  - python

Go experience

- Vitess & Go
- Go tips

Code base

- memcache, zookeeper and mysql clients
- bson protocol
- rpcplus
- connection pooling
- process management
- stats
- stream logging
- servers: vttablet, vtgate, zk proxy, vtctld
- tools: vtctl, mysqlctl, vtworker, zk browser
- Lines of code: ?

Expressive

- LRU Cache: 224 lines
- Connection pooler: 260 lines
- memcache client: 280 lines

Challenges

- Started in 2010: pre-1.0
- rpc performance
- cgo calls
- garbage collector
- network I/O
- runtime scheduler
- memory leaks

List in LRUCache

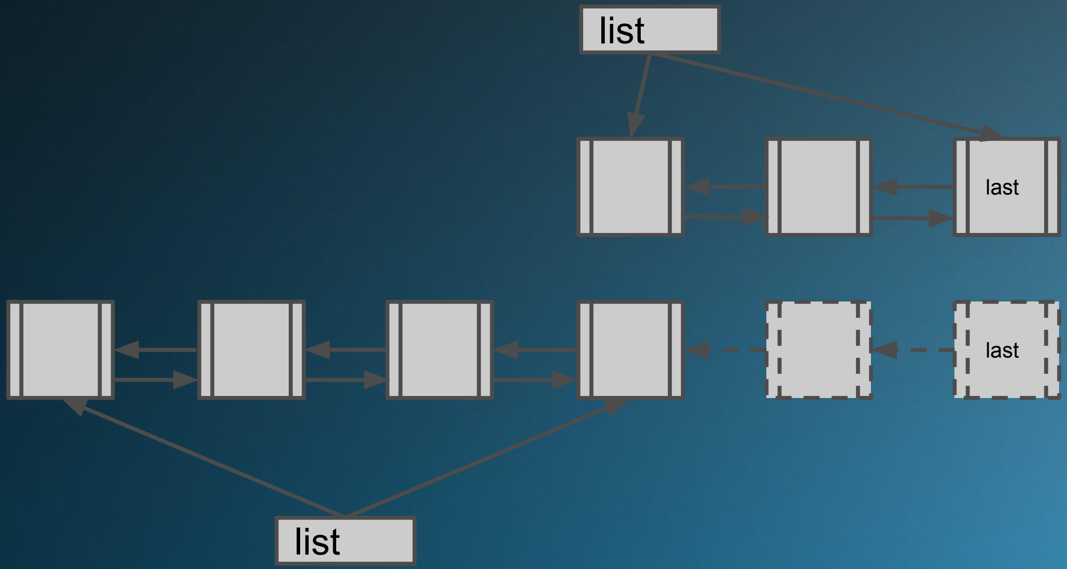

Go highlights

- Language
  - minimalist
  - interfaces
  - goroutines
  - channels & selects
  - closures
  - defers
  - type conversions
  - type switch
- Tools
  - compiler
  - libraries
  - cgo
  - /debug/pprof
  - /debug/vars
  - race detector
  - gofmt
  - goimports

Generics

- High level generics
  - Use interfaces
  - Closures can also be used
  - Requires little bit of forethought (struct->interface)
  - Not efficient for primitive types/operations
- Language defined generics
  - maps, slices & channels
- Other low level generics
  - Requires code generation

```
~/gotest> cat min.go
package min

func Min(v1, v2 Generic) Generic {
        if v1 < v2 {
                return v1
        }
        return v2
}
~/gotest> gofmt -r 'Generic -> int' min.go
package min

func Min(v1, v2 int) int {
        if v1 < v2 {
                return v1
        }
        return v2
}
~/go/src/pkg/container/list> gofmt -r 'interface{} -> int' list.go
```

C++ vs Go

- Performance: C++ generally wins (for now)
- Real-time systems: C++ wins
- Pretty much loses on everything else
  - Readability & maintainability
  - Type safety
  - Compiler speed
  - Platform independence
  - Debuggability
  - Null terminated strings

Python vs Go

- Expressibility
  - Python wins for small programs
  - Go comes out ahead for large programs/teams
- Libraries: Python wins (for now)
- Debuggability: Python wins
- Performance: Go wins

Pain points

- []byte vs string
- error vs panic
- channels are one way
- buffered vs. unbuffered channels
- GC for large footprints

Pitfalls - nil interface

```
func x() error {
  return y()
}

func y() *MyError {
  return nil
}
```

Is x() == nil?

Mitigation: Never return custom error types

Pitfalls - range vars

```
for _, url := range urls {
  go func() {
    fmt.Println(url)
  }()
}
```

How many urls get printed?

Mitigation(s):

```
for _, url := range urls {
  url := url
  go func() {
    fmt.Println(url)
  }()
}
```

```
for _, url := range urls {
  go func(url string) {
    fmt.Println(url)
  }(url)
}
```

Pitfalls - implicit conversions

```
var a int
var b interface{} = a
fmt.Printf(“Types: %T, %T\n”, a, b)
```

What gets printed?

- A conversion to interface is implicit
- An interface cannot contain another interface

Pitfalls - scoping

```
var a int
a, err := func() // only err new

var a int
if … {
  a, err := func() // a and err are new
}
```

Cool constructs

- Wrapping interfaces
- Methods on primitive types
- Closures are first class variables
- defer
- switch a.(type)

Wrapping interfaces

```
var accepts = sync2.AtomicInt64(0)

// CoutingListener tracks the total number accepted connections
type CountingListener struct {
  net.Listener
}

// Accept increments the ‘accepts’ counter before returning a connection.
func (l *CountingListener) Accept() (c net.Conn, err error) {
  c, err := l.Listener.Accept()
  if err != nil {
    return nil, err
  }
  accepts.Add(1)
  return c, nil
}
```

Methods on primitive types

```
type AtomicInt64 int64

func (i *AtomicInt64) Add(n int64) int64 {
  return atomic.AddInt64((*int64)(i), n)
}

func (i *AtomicInt64) Set(n int64) {
  atomic.StoreInt64((*int64)(i), n)
}

func (i *AtomicInt64) Get() int64 {
  return atomic.LoadInt64((*int64)(i))
}
```

Closures

```
import "http"

func foo() {
  ...
  http.HandleFunc(path, func(w http.ResponseWriter, r *http.Request) {
    myfunc(path, w, r)
  })
}
```

- Create them anywhere
- Use them anywhere
- maps, slices, function arguments, return variables, go calls, defers, ...

Generics using closures

```
func withRetry(action func(conn *Conn) error) error {
  for i := 0; i < 3; i++ {
    conn, err = GetConn()
    if err != nil {
      continue
    }
    err = action(conn)
    if err == nil {
      return nil
    }
  }
  return err
}
```

```
func Begin() error {
  return withRetry(func(conn *Conn) error {
    return conn.Begin()
  })
}

func Execute(query *Query) (*Result, error) {
  var r *Result
  err := withRetry(func(conn *Conn) error {
    var innerErr error
    r, innerErr = conn.Execute(query)
    return innerErr
  })
  return r, err
}
```

Closures (another example)

```
func foo() {
  mu.Lock()
  defer mu.Unlock()
  ...
  func() {
    mu.Unlock()
    defer mu.Lock()
    // Perform some unlocked ops here, like waiting
  }()
  // Resume locked operation here
}
```

switch on type

```
switch value.(type) {
case []byte:
  out = fmt.Sprintf("%q", value)
default:
  out = fmt.Sprintf("%v", value)
}
```

Reusable parts of vitess

- bson
- bytes2: ChunkedWriter
- cache: LRUCache
- cgzip: A faster gzip (wraps C library)
- flagutil: flags for string lists & maps
- hack: Unsafe (but efficient) casts

Reusable parts of vitess (contd)

- memcache: memcache client
- mysql: Low level mysql client
- netutil: Convenience ip & dns functions
- pools: Resource pools
- proc: Zero-downtime restart
- rpcplus: rpc+context+streaming

Reusable parts of vitess (contd)

- stats: advanced stats for expvar
- streamlog: non-blocking http logger
- sync2: nifty sync wrapper
- timer: time, with housekeeping conveniences

Vitess team

- Dev
  - Alain Jobart
  - Sugu Sougoumarane
  - Shruti Patil
  - Ric Szopa
  - Liang Guo
- Ops
  - Prashanth Labhane
  - Jojo Antonio
  - Dan Rogart
  - Prem Gogineni

We’re hiring!

Questions?

- Vitess: https://github.com/youtube/vitess
- Go: http://golang.org
